# Diminished neural responses predict enhanced intrinsic motivation and sensitivity to external incentive

**DOI:** 10.3758/s13415-014-0324-5

**Published:** 2014-10-28

**Authors:** Karen E. Marsden, Wei Ji Ma, Edward L. Deci, Richard M. Ryan, Pearl H. Chiu

**Affiliations:** 1 Baylor College of Medicine, Houston, TX USA; 2 Center for Neural Science and Department of Psychology, New York University, New York, NY USA; 3 Department of Clinical and Social Sciences in Psychology, Rochester, NY USA; 4 Virginia Tech Carilion Research Institute, 2 Riverside Circle, Roanoke, VA 24016 USA; 5 Department of Psychology, Virginia Tech, Blacksburg, VA USA

**Keywords:** Intrinsic motivation, Functional magnetic resonance imaging (fMRI), Internal and external incentives, Neural substrates, Behavioral performance

## Abstract

The duration and quality of human performance depend on both intrinsic motivation and external incentives. However, little is known about the neuroscientific basis of this interplay between internal and external motivators. Here, we used functional magnetic resonance imaging to examine the neural substrates of intrinsic motivation, operationalized as the free-choice time spent on a task when this was not required, and tested the neural and behavioral effects of external reward on intrinsic motivation. We found that increased duration of free-choice time was predicted by generally diminished neural responses in regions associated with cognitive and affective regulation. By comparison, the possibility of additional reward improved task accuracy, and specifically increased neural and behavioral responses following errors. Those individuals with the smallest neural responses associated with intrinsic motivation exhibited the greatest error-related neural enhancement under the external contingency of possible reward. Together, these data suggest that human performance is guided by a “tonic” and “phasic” relationship between the neural substrates of intrinsic motivation (tonic) and the impact of external incentives (phasic).

**Electronic supplementary material:**

The online version of this article (doi:10.3758/s13415-014-0324-5) contains supplementary material, which is available to authorized users.

The nature of human motivation has long intrigued scientists and practitioners in the areas of decision-making (Kahneman & Tversky, 1979), educational psychology (Dweck, 1986), artificial intelligence (Barto, Singh, & Chentanez, 2004; Kaplan & Oudeyer, 2007), economic behavior (Bénabou & Tirole, 2003; Camerer & Hogarth, 1999), and the pathophysiology and therapy of psychiatric illnesses (Der-Avakian & Markou, 2012; Miller & Rollnick, 2002). Data from these areas emphasize that human motivation derives from internal and external sources that operate on differing time scales, and that can complement or compete with one another to influence both the duration and quality of behavioral performance (Deci, Koestner, & Ryan, 1999; Dweck, 1986). However, understanding the neuroscientific basis of the interplay between internal and external motivators has been challenging, in part due to the use of varied, often subjective, measures of intrinsic motivation. Here, we operationalized intrinsic motivation as free-choice time spent on a task when task performance was not required (Deci et al., 1999); this metric guided our investigation of the neural substrates of intrinsic motivation and the neurobehavioral effects of external rewards.

A robust literature has indicated that actions are motivated by external rewards through a neural learning system in which decisions are reinforced by outcomes that are better (or worse) than expected (Montague, Dayan, & Sejnowski, 1996; Pessiglione, Seymour, Flandin, Dolan, & Frith, 2006; Schultz, Dayan, & Montague, 1997). These neural “prediction errors” guide learning and maximize an agent’s reward over time (Dayan & Balleine, 2002; Montague, King-Casas, & Cohen, 2006; Sutton & Barto, 1998), and anomalies in value-guided decisions have been implicated in psychiatric conditions including depression and substance dependence (Chiu & Deldin, 2007; Chiu, Lohrenz, & Montague, 2008). In contrast, very little is known about the neural substrates supporting intrinsic motivation, despite the central role of internal factors for guiding behavior (for a few studies that have reported neural responses to stimuli consistent with participants’ self-described internal motivation, see Bengtsson, Lau, & Passingham, 2009; Linke et al., 2010; Mizuno et al., 2008).

In general terms, actions are considered intrinsically motivated if an agent engages in behavior for its own sake, without attempting to attain external consequences (Barto et al., 2004; Kaplan & Oudeyer, 2007). In behavioral studies, intrinsic motivation has been linked to more positive self-reported experience (Leary, 2007), and from an evolutionary perspective, intrinsic motivation is thought to facilitate exploration and lead to knowledge or skills that confer a fitness advantage (Berlyne, 1966; Hebb, 1955). When tasks are not inherently interesting, external incentives may be offered to evoke performance (Camerer & Hogarth, 1999), and moderate external incentives can improve the behavioral output when rewards are operative (Lazear, 2000; Murayama, Matsumoto, Izuma, & Matsumoto, 2010), though performance decrements have been observed when incentives are large (Ariely, Gneezy, Loewenstein, & Mazar, 2009; Chib, De Martino, Shimojo, & O’Doherty, 2012). This phenomenon was explored in a provocative study showing that activity in neural regions involved in extrinsic reward valuation was excessively diminished when rewards were removed, and that the diminished neural activity paralleled decreases in intrinsic motivation (Murayama et al., 2010). Together, these data highlight that motivated behavior may be characterized on at least the axes of the duration and the quality of output, neither of which alone is sufficient for evolutionary fitness, but each of which may be influenced by both internal and external factors.

To examine the neural underpinnings of intrinsic motivation and the interplay with external incentives, we measured intrinsic motivation as the free-choice time spent on a word problem task beyond the expectations of the experimenter and informed a subgroup of participants that performance on the task would be rewarded (after Ryan & Deci, 2000). Using this paradigm and functional magnetic resonance imaging (fMRI), we report a network of brain regions in which diminished task-related activity predicted increased subsequent intrinsic motivation, and show that external contingencies enhanced behavioral performance and neural responses in these regions for task-critical events, particularly in the most intrinsically motivated participants.

## Materials and method

### Participants

A group of 43 right-handed, MRI-compatible male and female participants were recruited to participate in a neuroimaging study involving word problem solving. The participants were limited to native speakers of English due to the nature of the task, which required knowledge of common English phrases. All participants gave written informed consent and were paid for their participation. The study was approved by the Institutional Review Boards of Baylor College of Medicine and Virginia Tech. Three participants were excluded from the fMRI data analysis: one who moved excessively during the scan, and two whose data were contaminated by scanner artifacts. Thus, 40 individuals were included in the imaging analysis, and 43 were included in the behavioral analysis.

### Experimental session

#### Overview

**Fig. 1:**
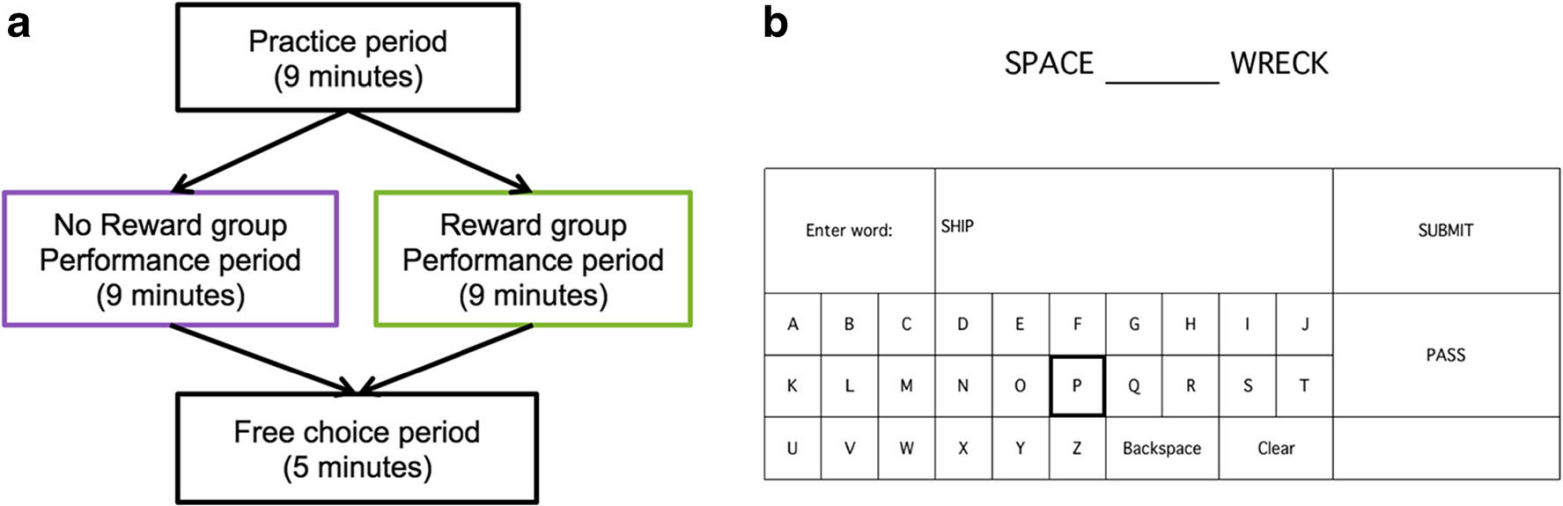
Schematic depictions of the experimental flow and the remote-associates word problems task. (a) Following a 9-min practice period in which participants practiced completing remote-associates word problems, participants were assigned to a “no-reward” or a “reward” group. During the performance period, all participants completed additional remote-associates word problems. The session ended with a 5-min free-choice period, during which they were given the options to complete more problems, turn off the screen, or read an archived digital newspaper. “Intrinsic motivation” was operationalized as the percentage of free-choice time spent completing word problems during this free-choice period. (b) For each word problem, participants were presented with two words and asked to complete the blank with another word that would form a separate common phrase or compound word with each of the two onscreen words. Participants typed their responses with an onscreen keyboard. Upon submitting an answer, participants were shown onscreen feedback indicating, for instance, “The most common answer is ‘ship’.” The word problem trial onsets were the events of interest in the present analysis

The experimental session was divided into three sections: a practice period, a performance period, and a “free-choice period” (see Fig. 1a for a schematic representation). Each section of the task was performed in the scanner, thus allowing consistency of procedures and timing between the sections across participants. In the scanner, bimanual two-button optical response boxes allowed the participants to navigate a cursor and submit responses on an onscreen keyboard (developed with Psychophysics Toolbox Version 3). All participants began with the practice period, during which they acclimated to unambiguous, single-solution, remote-associates-type word problems (Mednick & Mednick, 1967) and typing on the onscreen keyboard (Fig. 1b).

##### Remote-associates word problems

Remote-associate-type word problems (Bowden & Jung-Beeman, 2003; Mednick & Mednick, 1967) were used in each phase of the study. For each word problem, participants were presented with two words separated by a blank and were asked to complete the blank with another word that would form separate common phrases or compound words when it followed the first word and when it preceded the second word. For example, a solution to the problem “SUN _____ HOUSE” would be “LIGHT,” because “sunlight” and “lighthouse” are both common compound words. At the end of each trial, the most common solution was displayed (e.g., “The most common answer is ‘light’.”) Participants were instructed to try to complete the puzzle with the most common solution, and all responses were typed with the onscreen keyboard. Each remote-associates problem was displayed until one of three criteria was met: The participant submitted an answer, the participant chose to pass on the trial, or a 60-s time limit for the trial was reached. The feedback display time was jittered between 4 and 6 s. The items were presented in a random sequence, and no participant ever saw the same item more than once throughout the entire laboratory visit. Additional details about the remote-associates word problems are provided in the supplementary online materials.

##### Measuring intrinsic motivation and providing external incentive

Following the practice, and just prior to the performance period, participants were randomly assigned to one of two external incentive groups: “reward” or “no reward.” The “reward” participants (*N* = 21, eight males and 13 females; mean age = 24.6 years; *N* = 19 included in the imaging analysis) were instructed that upon task completion they would receive a monetary bonus based on their performance during the study, and the “no-reward” participants (*N* = 22, eight males and 14 females; mean age = 25.0 years; *N* = 21 included in the imaging analysis) were not offered any bonus. Participants then moved on to the 9-min performance period, which involved further remote-associates problems (mean per-puzzle solution time = 20.2 ± 5.3 s).

After the performance period, the study concluded with a 5-min free-choice period in which the following options were presented onscreen: (i) read a recently archived digital news site, (ii) clear the screen and wait for the experimenter, or (iii) continue with more remote-associates problems. During the free-choice period, participants could alternate freely among these three free-choice options. Upon task completion, participants’ intrinsic motivation to perform remote-associates problems was measured as the percentage of time that they chose to spend on word problems during the free-choice period. For categorical analyses, the participants who spent at least 50 % of this period on remote-associates problems were considered the “high-intrinsic” group. Prior to their departure, participants completed a posttask questionnaire that contained questions about the participants’ interest, perceived control, and perceived competence (items from the Intrinsic Motivation Inventory; Ryan & Deci, 2000) in completing the puzzles.

### Behavioral analyses

The behavioral analyses focused on intrinsic motivation and task performance under the external contingency of possible reward (or not). As we described above, intrinsic motivation was quantified as the percentage of time that each participant spent completing remote-associates problems during the free-choice period, and the incentive treatment was categorical, with a reward group and a no-reward group. The measures of performance included cumulative accuracy and response time, as well as accuracy and response time coded by the accuracy of the current and immediately subsequent trials (e.g., the accuracy and response time after correct or incorrect trials).

### Image acquisition and analyses

General linear model analyses of the imaging data were first conducted with the trial onset events to identify neural responses associated with intrinsic motivation, defined as free-choice time spent on the word puzzles when task performance was no longer required by the experimenter. The identified regions were then used in region-of-interest (ROI) analyses to examine the effects of possible reward (external incentive) on the neural substrates and associated behavior of intrinsic motivation. Given the high intercorrelation (discussed below in the Results) of the neural activity in the identified ROIs, we performed principal components analyses (PCAs) to identify a single metric for the shared variability in responses across the network of regions associated with intrinsic motivation. The first components derived from the PCAs were used in subsequent analyses relating neural activity to behavior. Additional details are provided below.

Continuous whole-brain imaging was implemented with a Siemens 3.0-T Trio scanner. Headphones reduced the noise from the scanner and allowed participants to hear the instructions. Participants were provided MRI-compatible glasses as needed. Initial high-resolution T1-weighted scans were acquired using an MP-RAGE sequence (Siemens). The functional run acquisition parameters were as follows: echoplanar imaging, gradient recalled echo, repetition time (TR) = 2,000 ms, echo time (TE) = 30 ms, flip angle = 90°, 64 × 64 matrix, slice thickness = 4 mm, field of view = 220 mm, 34 slices acquired hyperangulated 30° from the anteroposterior commissural line. Scanning yielded functional 3.4 × 3.4 × 4.0 mm voxels.

Data reduction and analysis of the images were performed using SPM8 (Wellcome Trust Centre for Neuroimaging, London, UK). Before analysis, the images were realigned, normalized, co-registered, segmented, and smoothed (6 × 6 × 6 mm). The imaging data for each participant were then fit to a general linear model that included six event regressors: trial onsets and answer reveals following correct and incorrect trials, and the first buttonpresses following trial onset for correct and incorrect trials. “Trial onset” was defined as the time at which the remote-associates problem was presented, and the “answer reveal” event was the time at which the solution to the problem was displayed. The onset events during the performance period were the primary focus of the present analyses. Events were modeled separately and convolved with a canonical hemodynamic response function. Two participants had no errors and were not included in the imaging analyses specific to errors. For the first- and second-level whole-brain analyses, statistical significance was defined as cluster size ≥5, *p* ≤ .001 uncorrected, and *p* ≤ .05, FDR-corrected for multiple comparisons.

The results of the low-intrinsic > high-intrinsic contrast identified six key regions that predicted intrinsic motivation for the word problems, regardless of extrinsic reward group; these regions were used in subsequent ROI analyses as being associated with intrinsic motivation. As a conservative estimate of ROI values, the average beta coefficients from the ROIs were extracted from the smaller of (1) the total cluster of significant activation in the anatomical region or (2) the 5-mm radius centered at the peak significant voxel in that region from the low-intrinsic > high-intrinsic contrast. Neural structures were defined anatomically using automated anatomical labeling (AAL), and ROIs were extracted from the total clusters of significant activation in bilateral amygdala and caudate, anterior cingulate cortex (ACC), parahippocampal gyrus (PHG), and anterior and posterior insula, as bilateral spheres around the peak voxels, all from the low-intrinsic > high-intrinsic contrast. The PHG ROI was adjusted to exclude any parts of the amygdala that might have been included in the spheres. Average beta coefficients for these ROIs were extracted and analyzed.

PCA (MATLAB R2010b) was conducted on the matrix comprising the beta coefficients for the trial onsets for each participant and each ROI, with columns being *z*-standardized to have a zero mean and unit variance. An analogous PCA was conducted on the matrix comprising the neural error reactivity values for each participant and each ROI, where neural error reactivity was defined for each participant as the difference in beta coefficients for the trial onsets immediately following errors and the beta coefficients for the trial onsets of the current trials on which errors occurred.

## Results

### Behavioral metrics of intrinsic motivation

**Fig. 2:**
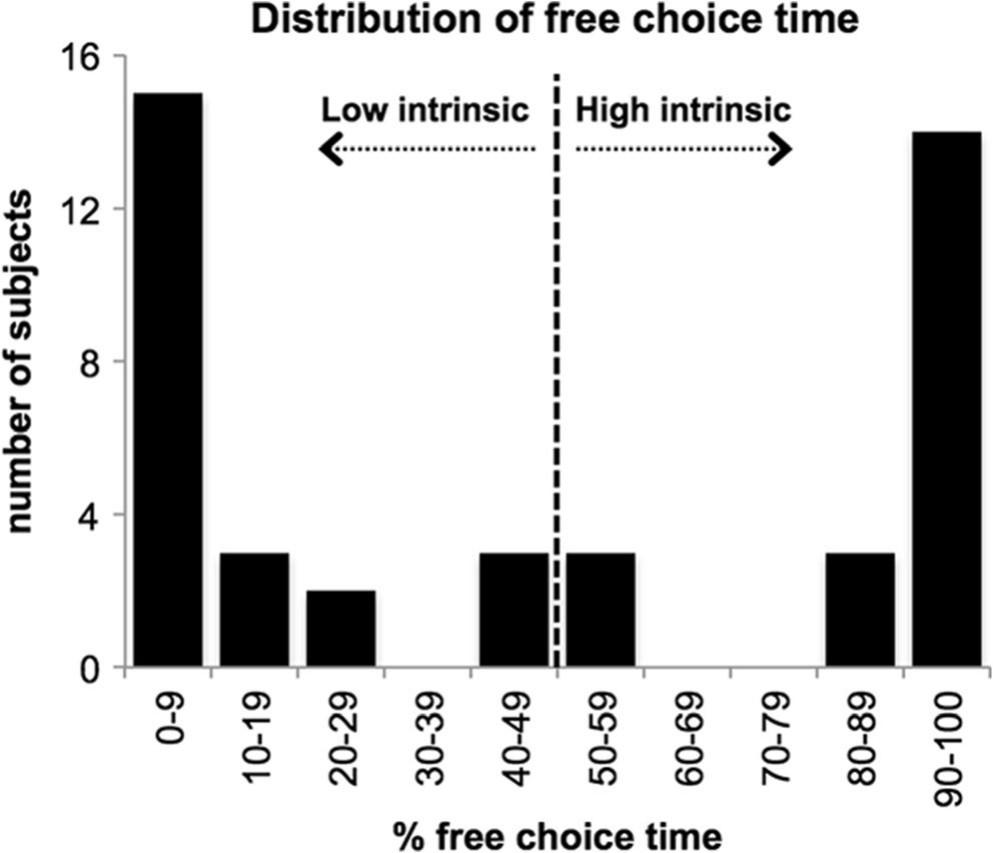
Distribution of participants’ intrinsic motivation to complete remote-associates word problems (*N* = 43). Each participant’s intrinsic motivation to complete word problems was quantified as the percentage of the total free-choice period spent on the problems. Participants exhibited a bimodal distribution of free-choice time. For our categorical analyses, those who spent at least 50 % of the free-choice period completing word problems were defined as being intrinsically motivated (“high intrinsic”), and those who spent less than 50 % of the free-choice period completing puzzles were considered to be low in intrinsic motivation to complete word problems (“low intrinsic”)

Intrinsic motivation was quantified following the experimental session as the percentage of free-choice time that participants spent on remote-associates word problems, with greater percentages of time working on problems indicating greater intrinsic motivation toward the word problems (for a review, see Deci et al., 1999). The distribution of free-choice time was bimodal rather than normal across all participants (*N* = 43), with roughly half of the participants spending at least 50 % free-choice time on remote-associates problems, and half spending less than 50 % (Fig. 2), consistent with previous work (Wiechman & Gurland, 2009). This 50 % criterion defined our “high” (*N* = 20) and “low” (*N* = 23) intrinsic motivation groups. Thus, participants in the high-intrinsic group, relative to the low-intrinsic group, allocated more time to attempting to complete word problems during the free-choice period (90.0 % and 11.2 %, respectively). The high- and low-intrinsic groups did not differ in number of problems completed, response time, age, gender composition, baseline mood assessed with the Positive and Negative Affect Schedule, or verbal IQ assessed with the Wechsler Test for Adult Reading (all two-tailed between-group comparison *p*s > .05). The two groups also performed equally well on the remote-associates problems during the performance period (*p* = .9) and were equally represented in the reward and no-reward conditions (nine of the 21 reward participants and 11 of the 22 no-reward participants were high-intrinsic; *χ* ^2^ = 0.22, n.s.). Posttask self-report questions that assessed the participants’ interest, perceived competence, and perceived control in completing the puzzles revealed that the two groups perceived the task as being equally enjoyable and interesting and themselves as being similarly competent (all between-group comparison *p*s > .1), and that the high-intrinsic group showed a trend toward greater perceived autonomy (i.e., “I did these word puzzles because I wanted to”; *p* = .054, two-tailed, uncorrected). Free-choice time was not related to behavioral accuracy (Spearman *r* = –.08, *p* = .6; see Fig. S1 in the supplemental materials).

### Neural predictors of intrinsic motivation

**Fig. 3:**
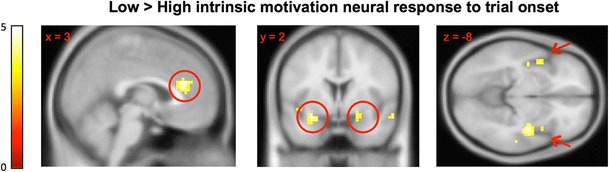
High-intrinsic-motivation participants showed diminished neural responses. The figure shows SPM8 *t* maps of neural responses to puzzle onset during the puzzle performance period, across the reward and no-reward groups [two-sample *t* test of the low-intrinsic-motivation > high-intrinsic-motivation groups: *t*(38) = 3.4, *p* = .0008 uncorrected, *p* = .05, FDR-corrected]. The six key regions identified in this contrast (anterior cingulate cortex, amygdala, anterior and posterior insula, parahippocampal gyrus, and caudate) were used in subsequent region-of-interest analyses as regions associated with intrinsic motivation

To identify neural regions associated with intrinsic motivation, we first compared blood oxygen level dependent (BOLD) activity in response to trial onsets during the performance period between the participants high and low in intrinsic motivation, regardless of extrinsic reward group. Specifically, a two-sample *t* test was conducted between all participants who spent at least 50 % (high intrinsic motivation) of the free-choice period on word problems and those who spent less than 50 % (low intrinsic motivation) of the free-choice period on the word problems. This whole-brain contrast identified a distinct pattern comprising six major brain structures: amygdala, caudate, ACC, PHG, anterior insula, and posterior insula [*t*(38) = 3.4, *p* ≤ .0008 uncorrected, *p* ≤ .05 FDR-corrected, Cohen’s *d* = 1.1; see Fig. 3]. In each of these regions, the high-intrinsic group exhibited significantly diminished neural responses relative to the low-intrinsic group (Fig. 3). The six primary regions identified in this contrast were used in subsequent ROI analyses as regions associated with diminished intrinsic motivation.

The BOLD responses to trial onsets were highly correlated among the six ROIs (range: *r* = .47 to .85), suggesting that responses in these regions were collectively related to intrinsic motivation for word problems. Thus, to identify a single metric accounting for the shared variability in responses across neural regions, we performed a PCA on the average trial-onset beta coefficients in each of these regions for each participant. The first principal component accounted for a majority (72.4 %) of the total variance in the six ROIs’ responses to trial onsets; each of the six ROIs’ beta coefficients was highly correlated with the PCA first-component scores (*r* from .78 to .89), suggesting that no single region’s response dominated the component. We thus used the first component scores from each participant's trial onset events as a composite measure of each participant’s neural response related to intrinsic motivation. No relationship was observed between participants’ behavioral accuracy and their trial-onset component scores (*r* = –.08, *p* = .63) or between accuracy and beta coefficients in any of the six regions (range: *r* = –.01 to –.26, all *p*s > .05).

### Effects of external incentive on neural substrates of intrinsic motivation

**Fig. 4:**
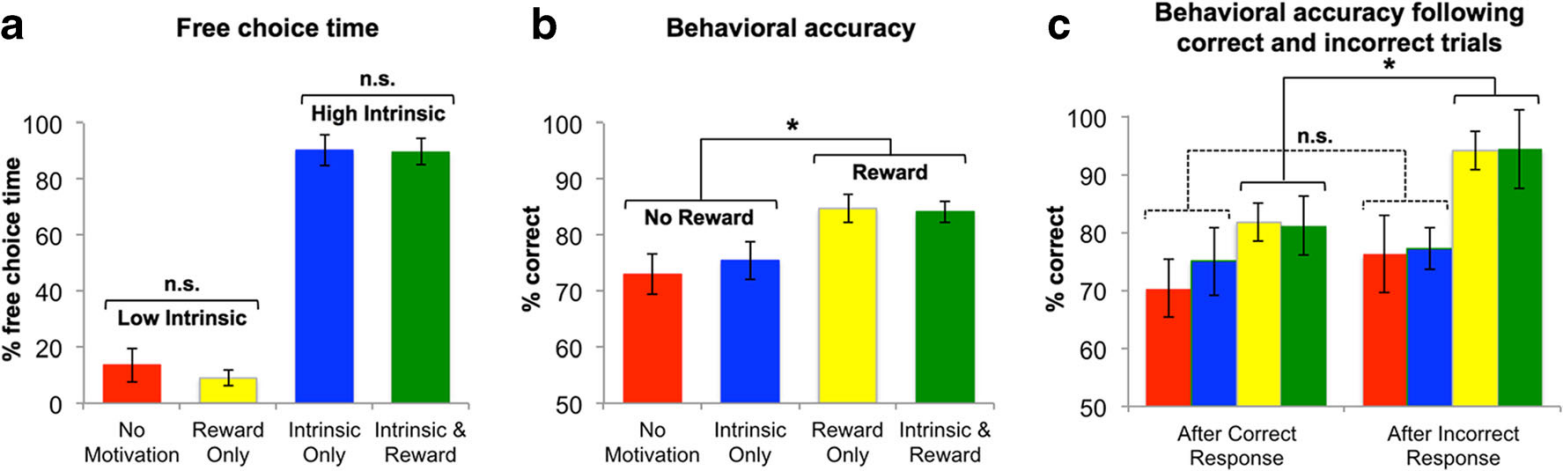
External reward incentive does not affect intrinsic motivation, but it enhances accuracy, particularly following incorrect responses. Participants in the “reward” group were instructed that they would receive a monetary bonus based on their final task performance. “High-intrinsic” participants were those who spent at least half of the free-choice time on remote-associates problems. This allowed us to identify four distinct subgroups, who were (i) low in intrinsic motivation and not offered reward (“No Motivation” bars), (ii) low in intrinsic motivation and offered an external reward for performance (“Reward Only” bars), (iii) intrinsically motivated without additional reward (“Intrinsic Only” bars), or (iv) both intrinsically motivated and offered the additional reward for performance (“Intrinsic & Reward” bars). (a) External reward incentive did not impact the amount of free-choice time that participants allocated to completing puzzles during the free-choice period [*F*(1, 21) = 0.0930, *p* = .48, and *F*(1, 18) = 0.009, *p* = .92, respectively, for the low- and high-intrinsic participants]. (b) In contrast, reward group participants showed greater accuracy during the puzzle performance period than did no-reward participants, regardless of intrinsic motivation status [*F*(1, 36) = 13.6, *p* = .001]. (c) The reward group exhibited enhanced accuracy following incorrect, relative to correct, responses [*F*(1, 20) = 19.44, *p* < .0001], regardless of intrinsic motivation, whereas the no-reward group showed no performance facilitation [*F*(1, 21) = 0.70, *p* = .41]. Error bars show standard errors of the means

To examine the impact of external contingencies on task performance and the neural substrates of intrinsic motivation, we assigned half of the participants to a “reward” condition in which they were instructed that they could receive a monetary bonus based on their final task performance. A univariate analysis of variance (ANOVA) with two between-group factors (Intrinsic, Reward) revealed that the potential for reward did not affect participants’ subsequent free-choice time spent on remote-associates problems [*F*(1, 42) = 0.25, *p* = .62; Fig. 4a]. In contrast, a significant effect of reward on accuracy was observed, such that participants who were offered reward for performance (“reward”) showed significantly greater accuracy than did those who were not (“no reward”), regardless of intrinsic motivation status [*F*(1, 39) = 11.33, *p* = .002, partial eta-squared (*η*_p_^2^) = .27; Fig. 4b].

The rich literature on reinforcement learning suggested the hypothesis that performance improvement occurs via prediction errors signaling that outcomes are “better or worse than expected,” and this led us to examine behavioral performance following correct and error trials. This analysis indicated that externally incentivized participants (reward) exhibited improved accuracy following error trials relative to following correct trials [*F*(1, 20) = 19.44, *p* = .0003, *η*_p_^2^ = .49], whereas those who did not receive external incentive (no reward) showed no performance boost [*F*(1, 21) = 0.70, *p* = .41, *η*_p_^2^ = .03; Fig. 4c]. To assess the nature of this behavioral enhancement, for each of the six ROIs identified to be related to intrinsic motivation, we computed a neural “error reactivity” measure, defined as the difference between the general linear model (GLM) beta coefficients for trial onsets immediately following errors and the beta coefficients for trial onsets on which errors occurred (beta for post-error-trial onset minus beta for error-trial onset; schematically depicted below in Fig. 6b). An analogous measure of “correct reactivity” was similarly defined as the difference between post-correct-trial onset beta values and correct-trial onset neural beta values. The BOLD responses in the six ROIs were highly correlated within the error-reactivity and correct-reactivity trials, respectively (range: *r* = .49 to .81), and we again performed PCA on the matrices comprising each participant’s neural error and correct reactivity in order to yield composite metrics of neural error reactivity and correct reactivity, respectively, for each participant. Again, the response in each ROI was significantly correlated with the first-component scores (range: *r* = .77 to .92), indicating that no single region likely dominated the components.

**Fig. 5:**
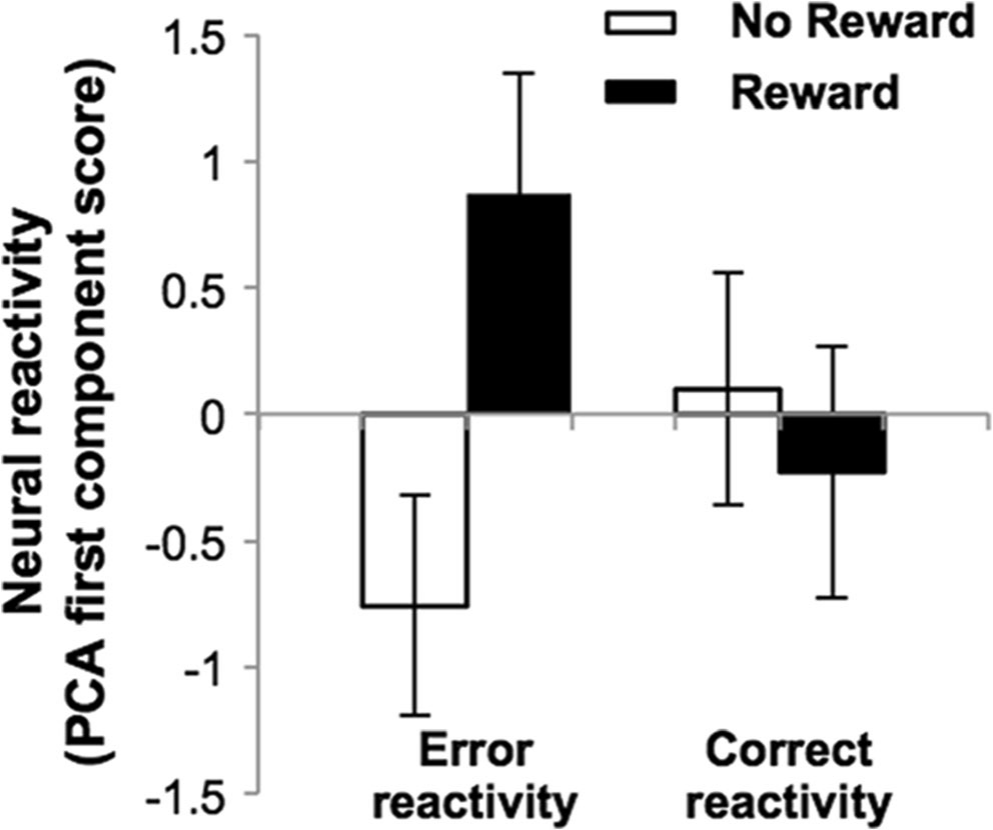
External incentive enhances neural responses following errors. For each participant, neural error (or correct) reactivity was quantified as the difference in neural responses (beta coefficients) to trial onsets immediately following errors (or correct responses) and trial onsets for the current trial on which errors (or correct responses) subsequently occurred. A repeated measures analysis of variance on the first neural PCA components for error and correct reactivities, with two between-group factors (Intrinsic: high, low; Reward: no reward, reward) and the within-group factor of Trial Type (error, correct), revealed a significant interaction [*F*(1, 33) = 4.29, *p* = .046, *η*_p_^2^ = .12] that was driven by enhanced neural error reactivity in the reward group [*F*(1, 36) = 6.26, *p* = .017, *η*_p_^2^ = .15]. No effects of reward or intrinsic motivation were observed for neural correct reactivity. Error bars show standard errors of the means

A 2 × 2 × 2 repeated measures ANOVA on the first neural PCA components with two between-group factors (Intrinsic: high, low; Reward: no reward, reward) and the within-group factor of Trial Type (error, correct) revealed a significant interaction [*F*(1, 33) = 4.29, *p* = .046, *η*_p_^2^ = .12] that was driven by differences in the reward and no-reward groups, specifically for error trials [*F*(1, 36) = 6.26, *p* = .017, *η*_p_^2^ = .15; Fig. 5]. No effects of reward or intrinsic motivation were observed for neural correct reactivity (see Fig. S2 for reward group effects by ROIs for correct and error reactivity). This increased neural reactivity in reward participants specifically to errors parallels the improved behavioral accuracy that this group showed following incorrect responses. Participants’ neural error reactivity scores regressed against behavioral accuracy following errors revealed that increased neural error reactivity significantly predicted improved accuracy following errors (*r* = .40, *p* = .013, two-tailed), suggesting that external contingencies selectively enhance effortful performance when needed—for example, following errors when improved performance is necessary to attain the reward. Post-hoc analyses suggest that the increased neural error reactivity conferred by external reward was evident in participants with high intrinsic motivation and not in the low-intrinsic group (see the supplemental materials and Fig. 3).

**Fig. 6:**
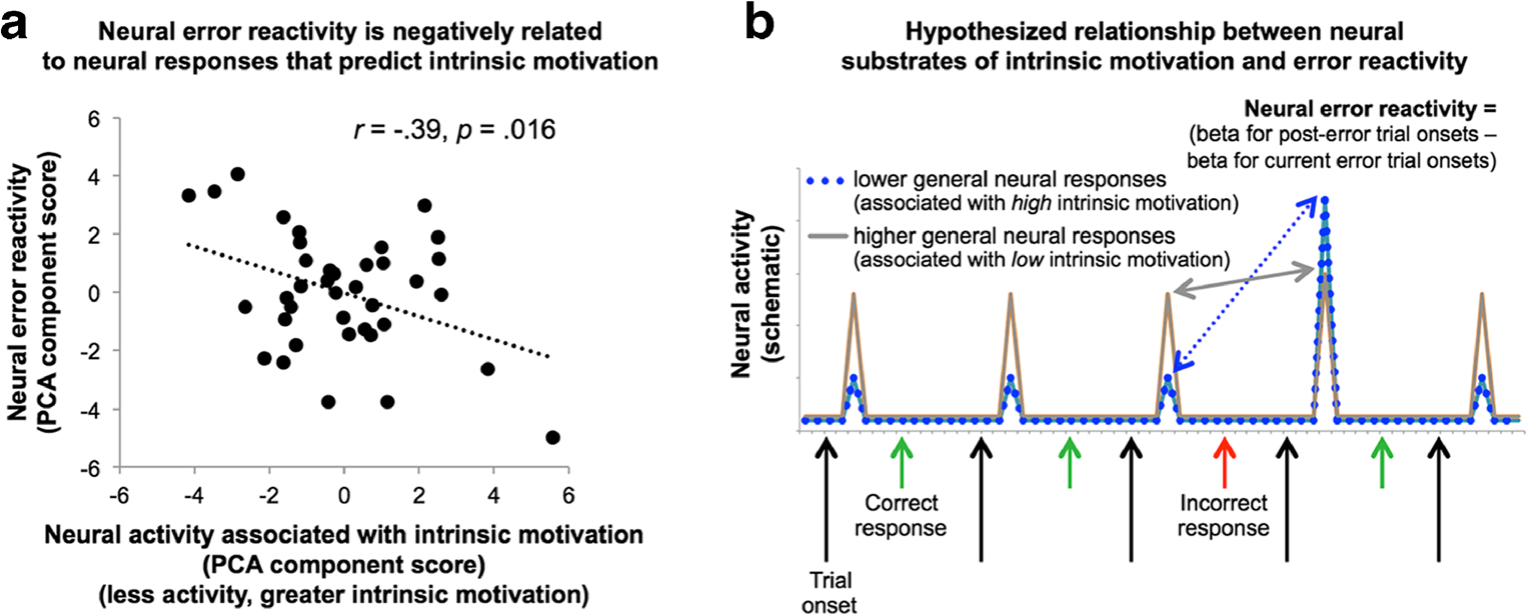
Neural responses associated with intrinsic motivation are negatively related to neural error reactivity. (a) Those who exhibit *smaller* BOLD responses to general trial onsets (i.e., those with higher intrinsic motivation) demonstrate *greater* BOLD responses in reacting to trials following errors, and vice versa (*r* = –.39, *p* = .016, two-tailed, between the component scores from the two PCAs). (b) A schematic diagram illustrates the hypothesis that high intrinsic motivation and its associated diminished general neural responses facilitate enhanced neural responses during critical task periods (here, posterror responding). The blue dotted line illustrates a low general neural response to trial onsets, which allows for a higher incremental neural response following errors under external incentives, as we posited for those with high intrinsic motivation. In comparison, the gray solid line illustrates a greater response to general trial onsets, which limits the neural reactivity to errors, as we have suggested for those with low intrinsic motivation. As is detailed throughout, the reward participants show enhanced neural error reactivity, and the data together suggest a “tonic” and “phasic” relationship between the neural substrates of intrinsic motivation (tonic) and the impact of external incentives (phasic). Behavioral response events were modeled in the neural general linear model analyses, but for simplicity are not illustrated here

Finally, to assess the interplay between neural substrates of intrinsic motivation and the neural error reactivity conferred by external incentive, we correlated participants’ first principal component scores of neural activity associated with general trial onsets (predictive of less intrinsic motivation) with the first principal component scores of neural error reactivity. This revealed a significant negative relationship (*r* = –.39, *p* = .016, two-tailed; see Fig. 6a), such that those who exhibited *smaller* responses to general trial onsets (greater intrinsic motivation) demonstrated *greater* neural responses to trials following errors. Moreover, those with smaller neural responses to general trial onsets (more intrinsic motivation) also showed shorter response times and increased accuracy following errors (*r* = .41, *p* = .009, and *r* = –.35, *p* = .027, respectively). These data support the hypothesis that intrinsic motivation and its associated diminished general neural responses facilitate enhanced neural responses and behavioral performance during critical task periods (here, during posterror responding). This possibility is schematically illustrated in Fig. 6b, wherein the dotted line illustrates a low general neural response to trial onsets, which allows for a higher incremental neural response following errors, and the solid line illustrates a greater response to general trial onsets, which limits the neural reactivity to errors.

Since the regions’ variance that loads onto the first PCA component of the intrinsic motivation analysis may differ from the variance of the regions loading onto the first component of the neural error reactivity scores, we regressed neural error reactivity responses in each of the six ROIs against the intrinsic motivation PCA first component scores to clarify the regions that might facilitate the negative relationship. This analysis indicated that the association between intrinsic motivation and the effects of external incentive were particularly prominent in the neural error reactivity responses in caudate (*r* = –.44, *p* = .006) and anterior cingulate (*r* = –.48, *p* = .002). This pattern is intriguing, since large literatures have highlighted the roles of caudate and anterior cingulate cortex in error signaling, particularly when task performance is incentivized (e.g., Kerns et al., 2004; Montague et al., 2006); this is further discussed below.

### Exploratory analyses

Although we have focused here on the neural substrates involved in intrinsic motivation and how external incentives affect processing in these regions, we also conducted several post-hoc exploratory analyses to address how differences in neural responding to external rewards may affect the relationship between external incentives and intrinsic motivation (detailed in the Supplementary Results in the supplemental materials). In brief summary, these analyses revealed greater activity specifically in right inferior frontal gyrus (IFG) in the no-reward > reward comparison at trial onsets (*p* < .001, uncorrected). We next compared (i) participants who ranked *above the median on both* intrinsic motivation and reward group neural responses (i.e., on the first component of the intrinsic motivation PCA and the beta coefficients extracted from the reward-related IFG ROI, respectively) and (ii) those who ranked *below the median on both* neural measures. On both free-choice time and behavioral accuracy after errors, the “low” neural response group showed optimal performance (greater free-choice time, plus greater behavioral accuracy following incorrect responses). IFG is typically activated during response inhibition (for a review, see Aron, Robbins, & Poldrack, 2014), and these data support the hypothesis that during task performance, reduced neural responses in regions associated with neurocognitive/affective regulation may be a biomarker for a subset of individuals who are both intrinsically motivated and sensitive to the performance boosts that external incentives confer following errors.

## Discussion

Taken together, these data identify neural predictors of intrinsic motivation and begin to clarify the dynamics between intrinsic motivation and external incentives. The regions in which less general task-related activity is associated with subsequent greater intrinsic motivation—amygdala and parahippocampal gyrus, anterior and posterior insula, anterior cingulate cortex, and caudate—have separately and collectively been implicated in affective regulation, cognitive control, and learning (Craig, 2009; Critchley, Wiens, Rotshtein, Öhman, & Dolan, 2004; Kerns et al., 2004; LeDoux, 2012; Leotti & Delgado, 2011; Montague et al., 2006; Sheth et al., 2012). Although our present paradigm is unable to parse out the specific contributions of these respective neural functions to intrinsic motivation, the consistent pattern of diminished neural activity in those with high intrinsic motivation fits with other data demonstrating that activating affective and cognitive control tends to diminish subsequent self-regulation and intrinsic motivation (Baumeister, Muraven, & Tice, 2000; Ryan & Deci, 2000). Following from this, in our case, decreased activation of neural affective/cognitive control may facilitate increased intrinsic motivation, here measured as task persistence.

In concert, the addition of a performance-based incentive enhanced participants’ neural and behavioral sensitivity to errors. Specifically, the reward participants did not show enhanced free-choice times, but did show enhanced neural reactivity to errors in the network of regions implicated in intrinsic motivation, and this reactivity for reward participants in turn predicted behavioral improvement on the trials subsequent to errors (see Laming, 1979, for a classic report of posterror behavioral improvement). These neural and behavioral data fit with models of reinforcement learning, which highlight the role of striatal regions, including caudate, in neural error signaling of outcomes that are better or worse than expected in learning, so as to maximize rewards over time (for reviews, see Dayan & Balleine, 2002; Montague et al., 2006). Extensive literatures also implicate the anterior cingulate cortex in posterror processing and behavioral adjustments (Kerns et al., 2004; Sheth et al., 2012), and the amygdala and insular cortices in regulating affective responses to and interoceptive awareness of errors (Craig, 2009; Critchley et al., 2004). Of note, the enhanced neural error reactivity evoked by external incentive was most prominent in those with the smallest neural responses associated with intrinsic motivation (i.e., the high-intrinsic group; see Fig. 6a and Fig. S3). Behaviorally, though, reward participants showed greater accuracy following errors (mean over 90 % correct), regardless of intrinsic motivation status. This overall high accuracy in reward participants suggests the possibility that a task with a higher “ceiling” may allow an even greater behavioral facilitation effect in high-intrinsic participants that matches the increased neural reactivity seen in this group, yielding individuals who perform with both duration and increased quality when appropriate extrinsic incentives are provided. This remains an intriguing topic for future investigation.

The present data suggest complementary neurobehavioral processes: Greater intrinsic motivation is associated with generally diminished neural responses that extend task engagement, whereas external contingencies enhance neural sensitivity and behavioral adjustments specifically to failure events. In particular, an overall state of dampened neurocognitive regulation, suggested by the lower general task-related neural responses in our high-intrinsic group, may facilitate enhanced neural and behavioral responses when needed, as in the case of errors, and when evoked by positive external contingencies for doing well. That is, in line with a “resource depletion” framework (Baumeister et al., 2000; Muraven, Shmueli, & Burkley, 2006; Vohs & Heatherton, 2000), it may be the case that individuals high in intrinsic motivation for an activity tend to “conserve resources,” which renders these neurocognitive resources available when they are needed during critical task periods. Consonant with this hypothesis, we observed a significant negative relationship between general neural responses to trial onsets and specific neural reactivity following errors, such that those who exhibited diminished responses to general trial onsets also demonstrated enhanced neural responses on trials following errors. The apparently distinct functions of the neural substrates of intrinsic motivation (associated with general task processing) and the neural impact of external contingencies (associated with task-related error events) are also notable for their consistency with molecular and computational model-based accounts of “tonic” and “phasic” neuromodulatory functions that subserve motivation and learning (including serotonergic, dopaminergic, and norepinephrine systems; Aston-Jones & Cohen, 2005; Barto et al., 2004; Daw, Kakade, & Dayan, 2002; Howe, Tierney, Sandberg, Phillips, & Graybiel, 2013; Montague et al., 2006).

### Future considerations

Human motivation derives from internal and external sources that can complement one another to influence both the duration and quality of behavioral performance, although under some conditions the influences may be antagonistic (Deci et al., 1999; Murayama et al., 2010). Indeed, external rewards have been related to impaired error processing in mood, anxiety, and substance-related disorders (Chiu & Deldin, 2007; Chiu et al., 2008; Paulus, Feinstein, Simmons, & Stein, 2004) and have been shown to undermine intrinsic motivation in some cases (Lepper, Greene, & Nisbett, 1973; Murayama et al., 2010), but not in others (Cooke et al., 2011; Kremer, Miguel, & Thornton, 2009; see Deci et al., 1999; Wiechman & Gurland, 2009, for a thorough discussion of these issues), thus emphasizing the need to consider both the benefits and the costs of manipulating external incentives. Also, we caution that the present data are unable to establish a causal relationship between neural responses and intrinsic motivation; that is, it may be the case that the highly intrinsically motivated conserve neural resources for use during critical events, or that activating neural control during general task performance diminishes intrinsic motivation. Additional studies will be needed to differentiate these possibilities. Finally, our findings of increased neural signaling and behavioral accuracy following error trials particularly among high-intrinsic + reward participants may be relevant to computational models of learning and theories of motivation used in computer intelligence (e.g., Dayan & Balleine, 2002; Kaplan & Oudeyer, 2007; Sutton & Barto, 1998). Future investigations that incorporate learning of stimulus–outcome contingencies and valuation of objective rewards with measures of intrinsic motivation will facilitate the use of formal learning models to test this possibility.

## Conclusions

Our data point to the potential importance of considering intrinsic motivation in error signaling under external incentives. The results indicate that greater intrinsic motivation, quantified here as free-choice time on a task, is predicted by lower general task-related neural activity, suggesting less tonic expenditure of neural resources in individuals high in intrinsic motivation. In comparison, external incentive improves task performance (here, accuracy) and yields increased phasic neural sensitivity to errors. This dissociation points to an interesting subset of individuals in the present data—those who evidenced high intrinsic motivation (low general task-related neural activity) and at the same time exhibited behavioral and neural benefits from the offer of external reward for good performance, suggesting the possibility of a biomarker for individuals who perform with an optimal combination of duration and quality when appropriate extrinsic incentives are provided. Future clarification of the neurocomputational mechanisms that underlie intrinsic motivation and the impact of external contingencies will inform our understanding of the dynamics through which internal and external factors influence performance in the laboratory, schoolroom, corporate office, and clinic.

## Electronic supplementary material

Below is the link to the electronic supplementary material.

Fig. S1 (PDF 765 kb)
