# Supplementary material for: Diminished neural responses predict enhanced intrinsic motivation and sensitivity to external incentive
Source: Cogn Affect Behav Neurosci. 2014 Oct 28;15(2):276–86. doi: 10.3758/s13415-014-0324-5 (PMC4412758; doi:10.3758/s13415-014-0324-5)
Supplement: Supplementary file 1 — (PDF 765 kb) [file 13415_2014_324_MOESM1_ESM.pdf]

Supplemental Material for

Diminished neural responses predict enhanced intrinsic motivation and sensitivity to external incentive

Karen E. Marsden[1], Wei Ji Ma[1], Edward L. Deci, Richard M. Ryan, Pearl H. Chiu[*]

[1] Authors contributed equally. * Address correspondence to: pearlchiu@vtc.vt.edu

Supplemental Experimental Procedures includes additional detail about the remote associates word problems and procedures implemented in this work.

Supplemental Results includes exploratory analyses further parsing the relationship between external incentives and intrinsic motivation.

Supplemental Data

- Figure S1 shows that duration of free choice time is not related to behavioral accuracy.
- Figure S2 shows that external incentive does not affect neural responses following correct responses.
- Figure S3 illustrates post-hoc analyses suggesting that the increased error reactivity conferred by external reward was evident in the High Intrinsic group and not in the Low Intrinsic group.
- Table S1 shows that increased duration of free-choice time is predicted by diminished neural response in regions associated with cognitive/affective regulation.

Supplemental Experimental Procedures

Remote associates word problems

Remote-associates-type word problems (Mednick and Mednick, 1967; Bowden and Jung-Beeman, 2003) were used in each phase of this study. Participants were presented with two words separated by a blank and were asked to produce a word that makes a common phrase or compound word both when it follows the first word and when it precedes the second word. For example, a solution to the problem "SUN _____ HOUSE" would be "LIGHT" because "sunlight" and "lighthouse" are both common words. Subjects were instructed to try to produce the most common solution. A behavioral pilot was conducted with 24 subjects to select the remote associates problems to be included in the study. 150 items were tested, and 126 single-solution problems were chosen to be included. The final problem set consisted of 126 problems, and the mean accuracy of the pilot participants on the final chosen problems was 75.1%. The mean interest rating by the pilot participants of the included problems was 5.26 (Likert scale from 1 to 7; not at all, to very interesting).

During the scanning session, participants were presented with one remote associates problem at a time and entered their response using an on-screen keyboard (**Figure 1b**). A four button box was used in the scanner to navigate the on-screen keyboard, and all subjects practiced "typing" on the keyboard before the practice period until they were comfortable with the controls. Each remote associates problem was displayed until one of three criteria was met: the participant entered and submitted an answer, the participant chose to pass on the trial, or the time limit for the trial was reached. From the time that a problem was presented, participants had 60 seconds to provide and

submit an answer. If the subject was in the process of typing when the 60 seconds ran out, the program allowed them to continue until either they ended the trial with either a submission or pass, or no buttons were pressed for five seconds. At the end of each trial, the most common solution was displayed (e.g., “The most common answer is ‘light’.”). Feedback display time was jittered between 4 to 6 seconds. The items were presented in a random sequence, and no participant ever saw the same item more than once throughout the entire laboratory visit.

Procedure

A schematic diagram of the experimental procedure is displayed in **Figure 1**. When participants arrived, informed consent was obtained, and the experimenter provided instructions about the remote associates problems as well as scanner safety instructions. Upon entering the scanner, subjects completed several sample problems to verify that they understood the instructions and could easily navigate the keyboard. Throughout the task, instructions were both displayed on screen and read to the subjects. Before the practice period, all subjects received the same instructions and were told that they would have first have the opportunity to practice and “get used to the puzzles”. After the practice period, individuals in the No Reward group were instructed that the main problem-solving period would begin and “Try to do your best and name as many of the common solutions as you can.” Those in the Reward group were further instructed they could receive a bonus of “up to $20” depending on performance, and the experimenter would “let you know how much you have earned at the end.” At the end of the performance period, the Reward group was told that they performed “well" on the puzzles and “earned an additional $15.” The No Reward group was also told that they performed “well,” but there was no mention of a reward. All participants then received the same instructions for the

remainder of the experimental session. The study concluded with the free-choice period with participants remaining in the scanner. Subjects were instructed that an anatomical scan was being collected and “While you’re waiting, you can do some more word problems, read recent articles from the New York Times, or just wait, and you can switch freely between these choices.” Subjects used the button boxes to alternate freely among these options. During this period, we recorded the time subjects spent completing remote associates items, reading the newspaper, or resting. All participants received $35 dollars for their participation, regardless of group membership.

Behavioral analyses

The behavioral analyses focused on intrinsic motivation and task performance under the external contingency of possible reward (or not). As described above, intrinsic motivation was operationalized as the percentage of time each participant spent completing remote associates problems during the free-choice period, and the incentive treatment was categorical as described above, with a Reward group and a No-Reward group. Measures of performance included cumulative accuracy and response time, and accuracy and response time after incorrect and correct responses, respectively. Accuracy was computed as the number of remote associates problems that a participant answered correctly in the performance period divided by the total number of problems that the subject completed during the performance period. Response time was defined as the amount of time between when a problem was first displayed to when the participant either submitted an answer, pressed the pass button, or timed out, thus representing the amount of time that the subject spent on a problem. For accuracy following incorrect and

correct responses, we coded the trials according to previous trials' accuracy and calculated accuracy on subsequent trials.

Supplemental Data

Relationship between intrinsic motivation principal components and free-choice time

As detailed in the main text, we performed a principal components analysis on the average trial-onset beta coefficients in each of 6 regions associated with intrinsic motivation for each participant. The first component scores from participants' trial onset events were used as a composite measure of each subject's neural response related to intrinsic motivation. As expected, participants' first PCA component scores were negatively related to free-choice time spent on word problems (Spearman *rho* = -0.46; indicating diminished task-related BOLD activity associated with subsequent increased intrinsic motivation). This pattern was evident within each individual ROI, such that free-choice time was correlated with neural responses in each of the six regions (r range -0.31 to -0.53); as expected, High and Low Intrinsic participants differed in trial-onset component scores ($F(1,39) = 16.86$, $p = 0.0002$; partial $\eta^2 = 0.31$).

Post-hoc analyses

Post-hoc analyses suggest that the increased neural error reactivity conferred by external reward is evident in participants with High Intrinsic motivation and not in the Low Intrinsic group ($F(1,18) = 8.49$, $p = 0.01$, partial $\eta^2 = 0.32$; $F(1,18) = 0.40$, $p = 0.48$, partial $\eta^2 = 0.03$ within High and Low Intrinsic participants, respectively; **Figure S3**).

Although the present paradigm was fully powered to detect significant neural and behavioral effects of intrinsic motivation (and did so), some of the follow up analyses may not have been sufficiently powered to detect significance at traditional alpha levels for interactions between intrinsic motivation and external rewards. For example, the data presented in **Figure S3**, while showing significant effects of Reward within the High and Low Intrinsic motivation groups, fell just short of traditional alpha values at the interaction level when two between group factors were included ($p = .12$). The effect size of the results shown in **Figure S3** fell in the small to medium range (partial $\eta^2 = 0.07$), suggesting a small interaction effect that would require a larger sample size to reach alpha = .05. Thus, in this case, power may be limiting our ability to detect significant interactions, and interpretation of the associated results should be made with caution. Future studies aimed to examine the effects suggested by the post-hoc analyses will be sufficiently powered.

Exploratory analyses

In this manuscript, we focus primarily on the regions involved in intrinsic motivation and how external incentives affected processing in these regions. Nonetheless, for the interested reader we have conducted several additional exploratory analyses to further parse the relationship between external incentives and intrinsic motivation.

These analyses revealed greater activity specifically in right inferior frontal gyrus (IFG) in the No Reward > Reward comparison at trial onsets ($p < .001$, uncorrected). This pattern suggested the hypothesis that individuals who showed greatest neural responses associated with both general intrinsic motivation *and* external incentives would show optimal performance on both

free-choice time (associated with intrinsic motivation) and accuracy after incorrect responses (associated with the Reward condition). To test this possibility, we divided participants into (i) those who ranked *above the median on both* intrinsic motivation and Reward group neural responses (first component of the intrinsic motivation PCA and beta coefficients extracted from the Reward-related IFG ROI, respectively) and (ii) those who ranked *below the median* on *both* neural measures. This classification yielded 14 participants below the median on both external reward and intrinsic motivation neural responses and 13 participants above the median on both responses (recall that the total N = 40; 13 participants were discordant on the intrinsic and extrinsic motivation neural measures). Univariate ANOVAs revealed that the High and Low "Neural Intrinsic Motivation response + Neural Reward response" groups differed significantly on both free choice time ($F(1,25) = 4.84$, $p = .037$, partial $\eta^2 = 0.31$) and behavioral accuracy following incorrect responses ($F(1,25) = 6.25$, $p = .019$, partial $\eta^2 = 0.2$). For both comparisons, the 'Low' neural response group showed optimal performance (greater free choice time, and greater behavioral accuracy following incorrect responses. As detailed in the main text, *diminished* neural responses during task performance predicts increased intrinsic motivation; the additional No Reward > Reward comparison identified here shows that diminished activity in IFG (typically activated during response inhibition; Aron et al., 2014) distinguishes the Reward group. Together, these data suggest that during task performance, *reduced* neural responses in regions associated with neurocognitive/affective regulation may be a biomarker (e.g., the 'brakes are off') for those who are both intrinsically motivated and sensitive to the performance boosts that external incentives confer.

To address this possibility, we generated a composite metric of general neural responses to intrinsic and extrinsic motivation for each participant (the sum of the beta coefficient from the Reward IFG ROI and the first component of the intrinsic motivation PCA); this metric showed a significant negative correlation with neural error reactivity (r = -.61, p = .0001). The negative relationship supports both the possibility of a 'composite biomarker' and our hypothesis (discussed in the manuscript) that diminished general neural activation facilitates enhanced neural and behavioral responses during critical task periods (here, post-error responding).

Although the present paradigm was *not* designed to evaluate the neural effects of obtained rewards (our Reward treatment simply informed participants they would be paid based on performance at the end of the lab visit), the results obtained in these exploratory analyses are intriguing and warrant future fully powered examinations of the interactive (and dynamic) effects between internal motivation and external incentives

Supplemental Figures

Supplemental Figure S1:

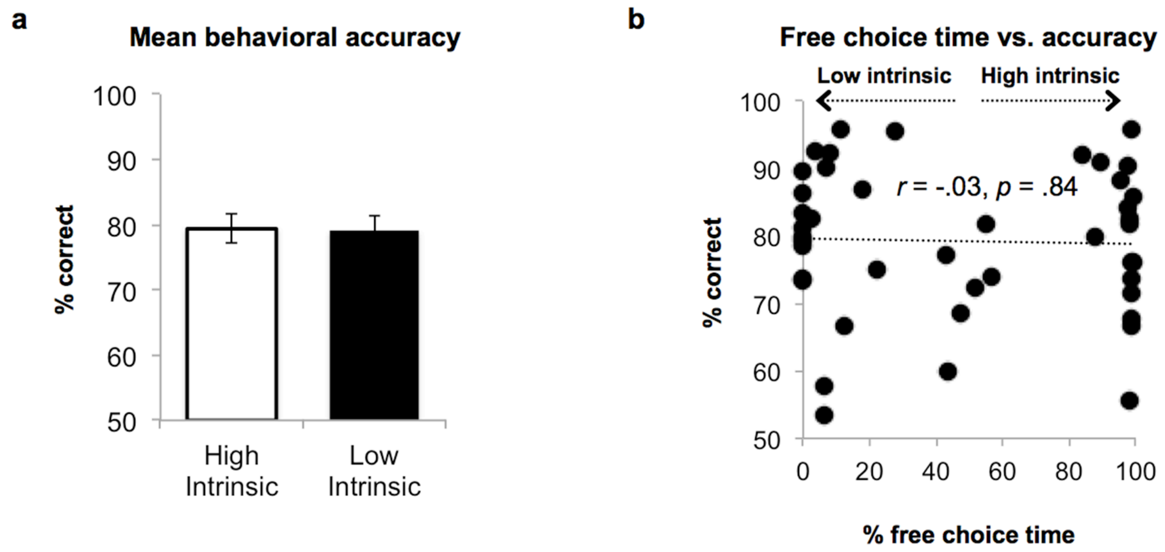

Figure S1. Duration of free-choice time was not related to task accuracy. (a) Participants who were intrinsically motivated toward the word problems ("High Intrinsic"), relative to those who were not ("Low Intrinsic"), did not differ in behavioral accuracy on the puzzles during the performance period (p = 0.94). Error bars represent standard error of the mean. **(b)** Intrinsic motivation was not correlated with behavioral accuracy.

Supplemental Figure S2:

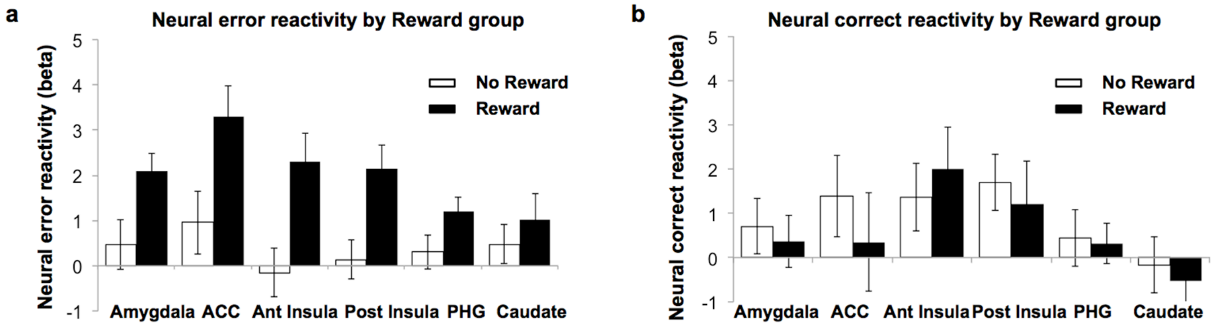

Figure S2. External incentive enhances neural reactivity following errors and does not affect neural reactivity following correct responses. For each participant, neural error (or correct) reactivity was quantified as the difference in neural response (beta coefficients) for trial onsets immediately following errors (or correct responses) and trial onsets for the current trial on which errors (or correct responses) subsequently occurred. Repeated measures ANOVAs examining Reward and No Reward participants' neural reactivity in the six ROIs implicated in intrinsic motivation (main text, **Figure 3a**) reveals (a) enhanced neural error reactivity in the Reward group ($F(1,36) = 6.42$, $p = .016$) and (b) no effect of Reward on neural correct reactivity ($F(1,37) = 0.10$; $p = .75$).

Supplemental Figure S3:

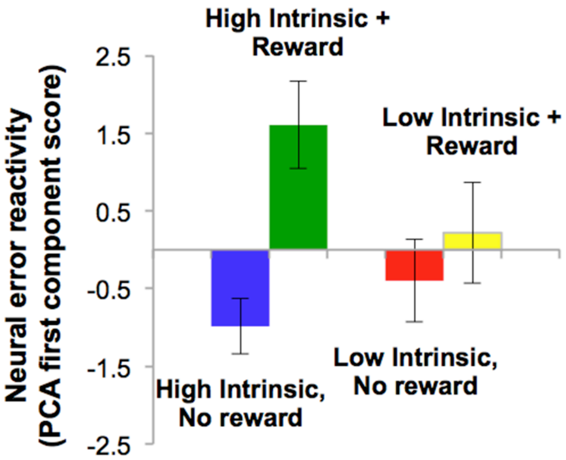

Figure S3. External incentive enhances neural responses following errors, particularly in High Intrinsic participants. Post-hoc analyses suggest that the increased neural error reactivity conferred by external reward was evident specifically in participants with high intrinsic motivation and absent in the low intrinsic group ($F(1,17) = 8.49$, $p = 0.01$; $F(1,17) = 0.40$, $p = 0.54$ within High and Low Intrinsic participants, respectively). Two participants made no errors and were thus not included in these analyses (leaving $N = 19$ in both the High and Low Intrinsic groups). Error bars show standard error of the mean.

Table S1. Increased neural activation during task performance predicts diminished subsequent free-choice time. In regions-of-interest associated with intrinsic motivation, neural responses to trial-onsets are negatively correlated with subsequent free-choice time. Two-tailed significance values and coordinates of maximal activation are listed.

Region-of-interest identified from Low Intrinsic > High intrinsic contrast (Figure 3)	Correlation of beta coefficient with free-choice time	*p* (two-tailed)	Coordinates of peak activation (MNI)
amygdala	-0.53	≤ 0.001	(-18, -4, -14)
parahippocampal gyrus	-0.49	0.001	(36, -10, -26)
anterior cingulate cortex	-0.43	0.006	(6, 32, 22)
anterior insula	-0.41	0.009	(42, 8, -2)
posterior insula	-0.47	0.002	(-48, -10, 4)
caudate	-0.31	0.05	(12, 8, -8)
